# Progressive Hypertrophic Genital Herpes in an HIV-Infected Woman despite Immune Recovery on Antiretroviral Therapy

**DOI:** 10.1155/2008/592532

**Published:** 2008-09-04

**Authors:** Mark H. Yudin, Rupert Kaul

**Affiliations:** ^1^Department of Obstetrics and Gynecology, St. Michael's Hospital, Toronto, ON, Canada M5B 1W8; ^2^Department of Obstetrics and Gynecology (MY), University of Toronto, ON, Canada M5G 1L4; ^3^Department of Medicine (RK), University of Toronto, ON, Canada M5G 1L4; ^4^Department of Medicine, University Health Network, Toronto, ON, Canada M5G 2C4

## Abstract

Most HIV-infected individuals are coinfected by *Herpes simplex virus* type 2 (HSV-2). HSV-2 reactivates more frequently in HIV-coinfected individuals with advanced immunosuppression, and may have very unusual clinical presentations, including hypertrophic genital lesions. We report the case of a progressive, hypertrophic HSV-2 lesion in an HIV-coinfected woman, despite near-complete immune restoration on antiretroviral therapy for up to three years. In this case, there was prompt response to topical imiquimod. The immunopathogenesis and clinical presentation of HSV-2 disease in HIV-coinfected individuals are reviewed, with a focus on potential mechanisms for persistent disease despite apparent immune reconstitution. HIV-infected individuals and their care providers should be aware that HSV-2 may cause atypical disease even in the context of near-comlpete immune reconstitution on HAART.

## 1. INTRODUCTION

Herpes simplex virus type 2 (HSV-2) is one of the most prevalent sexually transmitted infections worldwide [1]. HSV-2 is extremely common in countries where HIV is endemic, and coinfects over 75% of HIV-infected individuals living in or originating from these countries, but the HSV-2 seroprevalence is also over 50% in HIV-infected individuals from the developed world [2, 3]. Atypical or unusual manifestations of genital herpes are not uncommon in HIV-coinfected individuals, and persistent anogenital lesions due to HSV-2 were among the first opportunistic infections described in those with the acquired immunodeficiency syndrome (AIDS) [4]. Opportunistic infections may transiently worsen during the period of immune reconstitution following the initiation of highly active antiretroviral therapy (HAART), a phenomenon known as immune reconstitution inflammatory syndrome (IRIS), with HSV-2 reactivation accounting for up to half of IRIS cases [5]. However, the median time from initiating HAART to developing IRIS is three months, and IRIS is rare once stable immune reconstitution has been achieved [5]. Antiviral-resistant HSV is uncommon, although more frequently encountered among immunocompromised individuals compared to immunocompetent persons [6].

Hypertrophic anogenital lesions are a rare complication of HSV-2 in HIV-coinfected men and women [7–10], generally occurring in the context of significant immune deficiency or during IRIS [11], and these lesions may be very difficult to diagnose and treat. We present the case of a 35 year-old, HIV-infected woman with a recalcitrant hypertrophic vulvar lesion due to HSV-2. The development and prolonged persistence of this lesion after near-complete immune reconstitution on HAART implies that there are significant residual defects in host HSV-2 immune control, and that these may have important clinical implications for patients and their care providers.

## 2. CASE REPORT

The patient was a 34-year old, asymptomatic Zimbabwe-born woman with a positive HIV IgG ELISA on immigration screening in 2002. There was no prior history of sexually transmitted infections, genital or perianal ulceration, and syphilis serology was negative. Her absolute CD4+ T cell count at diagnosis was 156/mm^3^, with an HIV-1 RNA plasma viral load of more than 100 000 copies/mL. Antiretroviral therapy was initiated one month later, in October 2002, with combivir one tablet twice daily and nevirapine 200 mg twice daily, together with trimethoprim/sulfamethoxazole as primary prophylaxis against *P. jiroveci* pneumonia. By January 2003, her CD4+ T cell count had increased to almost 500/mm^3^, and her HIV viral load remained persistently undetectable after March 2003, at which point trimethoprim/sulfamethoxazole was discontinued. In April 2003, she noted multiple painful papules on the left labia, with subsequent superficial ulceration. She had no reported sexual contacts for over a year. Her family physician prescribed empiric therapy with acyclovir 400 mg three times daily and keflex 500 mg three times daily, followed by oral valacyclovir 500 mg twice daily.

**Figure 1:**
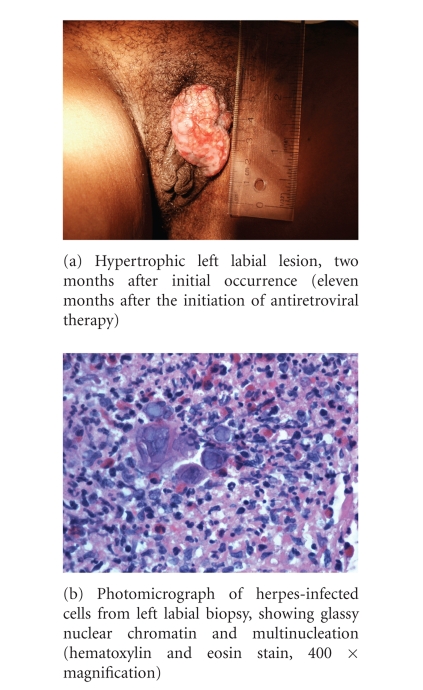
Clinical and microscopic appearance of hypertrophic HSV-2 lesion.

In July 2003, nine months after starting HAART, the shallow ulcerations had resolved but a pruritic 1 × 3 cm granulomatous lesion developed on the left labia. This was associated with surrounding tissue edema, and shotty left inguinal lymphadenopathy, and progressed over the subsequent two months (Figure 1(a)). There was no response to azithromycin 1 gram weekly for 4 weeks, as empiric therapy for granuloma inguinale. Both a superficial swab and a punch biopsy of the hypertrophic lesion demonstrated HSV-2, with glassy nuclear chromatin, multinucleation and surrounding severe acute and chronic inflammation (Figure 1(b)). Topical therapy was initiated with trifluridine, together with oral valacyclovir 1 gram twice daily. There was no clinical response, although valacyclovir discontinuation was followed by an outbreak of painful, scattered shallow ulcerations that were culture positive for HSV-2. From November 2003 and December 2005 she was treated with 4 one-month courses of intravenous foscarnet; despite a near total clinical response to the first course, the response waned progressively and the fourth course was complicated by *Staphylococcus aureus* cellulitis at the line site. Lesion cultures were repeatedly positive for HSV-2, and a repeat labial biopsy in February 2006 showed only chronic granulation tissue. There was no response to a trial of topical protopic (tacrolimus) in August 2006.

In September 2006 there was a prompt clinical response to topical imiquimod 5%, applied 3 times weekly, with resolution of the lesion within eight weeks. She remains asymptomatic on oral valacyclovir 1 gram twice daily combined with topical imiquimod 5% as needed (approximately one topical application every two weeks) with minor residual labial scarring.

## 3. DISCUSSION

We have presented a case of an African female patient with recalcitrant hypertrophic genital HSV-2, which was clinically resistant to standard antiviral therapy and to intravenous foscarnet, but which responded promptly to topical 5% imiquimod. An initial “typical” genital herpes outbreak occurred in the context of rapid immune recovery, and was quite compatible with IRIS, as has been described in Ugandan men starting therapy [11]. However, while this outbreak responded to standard herpes therapy, the hypertrophic genital HSV-2 lesion developed and persisted despite near-complete immune recovery for up to three years. The rapid clinical response that was seen to topical imiquimod, an agonist of Toll-like receptor 7 (TLR7) that boosts both host innate and adaptive antiviral immunity [12], strongly implies that there were clinically significant defects in host antiherpes immunity despite HAART-induced immune recovery.

Hypertrophic or squamoproliferative anogenital lesions in HIV-positive individuals or those with AIDS are uncommon and can pose a diagnostic dilemma. The most likely cause of such lesions is either neoplasia or infection, although the differential diagnosis can be wide [13]. It may be difficult to determine the cause of a lesion based on appearance alone, and the sensitivity of various diagnostic tests can be affected by the reactive changes present in these lesions [10]. Further, small biopsies may be inconclusive or provide misleading information [8]. It may be necessary to employ repeat biopsies and viral cultures in cases suspicious for HSV. In our patient, the first biopsy of the lesion in 2003 was positive for HSV-2, but a second biopsy performed after the lesion had recurred in 2006 was negative for HSV-1, HSV-2, cytomegalovirus (CMV), and spirochetes using immunohistochemical special stains, showing only chronic granulation tissue present. This reinforces that it may be difficult to demonstrate virus even in a generous biopsy specimen, especially in the presence of chronic, extensive granulation tissue. Because this second biopsy was negative for HSV, it is possible that HSV reactivation occurred within this proliferative mass, rather than causing it. However, it is more likely that HSV was in fact the etiologic agent responsible for the progression.

The pathogenesis of proliferative and hypertrophic HSV lesions in the immunocompromised patient population is poorly understood. It is thought to be a reflection of the increased duration of the disease course rather than any inherent change in the pathogenicity of the HSV-2 itself [14]. Early reports hypothesized that immune dysfunction secondary to HIV, perhaps mediated by T-helper type 2 cytokines, might result in the epidermal hyperplasia [15]. Whatever the etiology of these lesions, their presence has been linked to immune function. In HIV-infected individuals, HSV-2 infection is associated with an increased number and size of genital lesions relative to immunocompetent individuals [16]. Vesicles and ulcers are typically more necrotic, painful, and heal more slowly [17]. Finally, as CD4 cell count drops and immune status worsens, recurrent outbreaks increase in frequency and severity [18].

Underlying the increased severity of HSV-2 disease in HIV-coinfected patients are defects in herpes-specific immunity. In a recent report of herpes simplex vegetans in an HIV-infected individual with severe CD4+ T cell depletion [19], a specific defect was demonstrated in the production of type I interferons by plasmacytoid dendritic cells in response to HSV. Dendritic cells within the epithelium of the genital tract itself are important in mediating protective immunity against HSV [20], but these cells are dramatically depleted in the genital mucosa of HIV-infected individuals [21]. Finally, HIV infection may be associated with impaired HSV-2-specific CD8+ T cell responses, although these defects improve progressively after starting HAART [22]. In contrast, our patient had a normal CD4 count, an undetectable viral load, and had been on HAART for six months prior to the development of her lesions. The pathogenesis of progressive hypertrophic HSV-2 in this context, where such immune defects would be expected to have improved substantially, is not clear, although a similar case has been reported of a rapidly growing and recurrent genital mass in an HIV-infected woman on HAART with a CD4 T cell count >500/mm^3^ [7].

Immunocompromised individuals are more likely to be infected with HSV-2 that is resistant to antiviral medications [6]. Resistance to acyclovir is usually associated with resistance to the other nucleoside analog drugs, including famciclovir and valacyclovir [9]. In such cases, foscarnet or topical cidofovir may be useful, since they have different mechanisms of action [23]. Unfortunately, antiviral resistance testing was not available for our patient, although the prompt response of her “classical” ulcerative genital herpes outbreak to both acyclovir and valacyclovir, with rapid recurrence when suppressive treatment was stopped, suggested drug sensitivity. However, the hypertrophic lesion was clinically resistant to multiple courses of famciclovir and valacyclovir, as well as to later courses of intravenous foscarnet. Ultimately, there was a rapid and complete response to topical 5% imiquimod cream. Others have reported varied success with the use of imiquimod for HSV-2 therapy [19, 24], and this drug should be considered in the armamentarium for resistant and recurrent genital HSV-2 in HIV-infected individuals. Thalidomide has also been described as useful in HIV-positive patients with hypertrophic lesions, although this drug was not used in our case [25].

While uncommon, hypertrophic or squamoproliferative HSV disease in the HIV-positive population can be a very challenging clinical condition. It is painful, disfiguring, and difficult to diagnose and treat. As demonstrated by our patient, it can be seen in individuals anywhere along the spectrum of immune dysfunction. If clinically suspected, repeated attempts at diagnosis with viral cultures and biopsies are warranted. These lesions are often resistant to first-line antiviral treatment, and may require less commonly used therapies such as foscarnet, cidofovir, imiquimod, or thalidomide. In our case, imiquimod eventually resulted in a good clinical outcome.
